# Lipotoxicity-induced mtDNA release promotes diabetic cardiomyopathy by activating the cGAS-STING pathway in obesity-related diabetes

**DOI:** 10.1007/s10565-021-09692-z

**Published:** 2022-03-02

**Authors:** Xiu Mei Ma, Kang Geng, Betty Yuen-Kwan Law, Peng Wang, Yue Li Pu, Qing Chen, Hui Wen Xu, Xiao Zhen Tan, Zong Zhe Jiang, Yong Xu

**Affiliations:** 1 Faculty of Chinese Medicine, Macau University of Science and Technology, Avenida Wai Long, Taipa, Macau People’s Republic of China; 2 State Key Laboratory of Quality Research in Chinese Medicine (Macau University of Science and Technology), Avenida Wai Long, Taipa, Macau People’s Republic of China; 3 Department of Endocrinology and Metabolism, The Affiliated Hospital of Southwest Medical University, Luzhou, Sichuan 646000 People’s Republic of China; 4 Metabolic Vascular Diseases Key Laboratory of Sichuan Province, Luzhou, Sichuan 646000 People’s Republic of China; 5 Sichuan Clinical Research Center for Nephropathy, Luzhou, Sichuan 646000 People’s Republic of China; 6 Department of Plastic and Burn Surgery, The Affiliated Hospital of Southwest Medical University, Luzhou, Sichuan 646000 People’s Republic of China

**Keywords:** Lipotoxicity, mtDNA release, cGAS-STING, Diabetic cardiomyopathy

## Abstract

Diabetic cardiomyopathy (DCM) is characterized by lipid accumulation, mitochondrial dysfunction, and aseptic inflammatory activation. Mitochondria-derived cytosolic DNA has been reported to induce inflammation by activating cyclic GMP-AMP synthase (cGAS)/the stimulator of interferon genes (STING) pathway in the adipose, liver, and kidney tissues. However, the role of cytosolic mtDNA in the progression of DCM is unclear. In this study, with an obesity-related DCM mouse model established by feeding db/db mice with a high-fat diet (HFD), we observed increased mtDNA in the cytosol and activated cGAS-STING signaling pathway during DCM, as well as the downstream targets, IRF3, NF-κB, IL-18, and IL-1β. In a further study with a palmitic acid (PA)-induced lipotoxic cell model established in H9C2 cells, we revealed that the cytosolic mtDNA was the result of PA-induced overproduction of mitochondrial ROS, which also led to the activation of the cGAS/STING system and its downstream targets. Notably, treatment of extracted mtDNA alone was sufficient to activate the cGAS-STING signaling pathway in cultured H9C2 cells. Besides, both knockdown of STING in PA-induced H9C2 cells and inhibition of STING by C-176 injection in the DCM mouse model could remarkably block the inflammation and apoptosis of cardiomyocytes. In conclusion, our study elucidated the critical role of cytosolic mtDNA-induced cGAS-STING activation in the pathogenesis of obesity-related DCM and provided preclinical validation for using a STING inhibitor as a new potential therapeutic strategy for the treatment of DCM.

**Graphical abstract:**

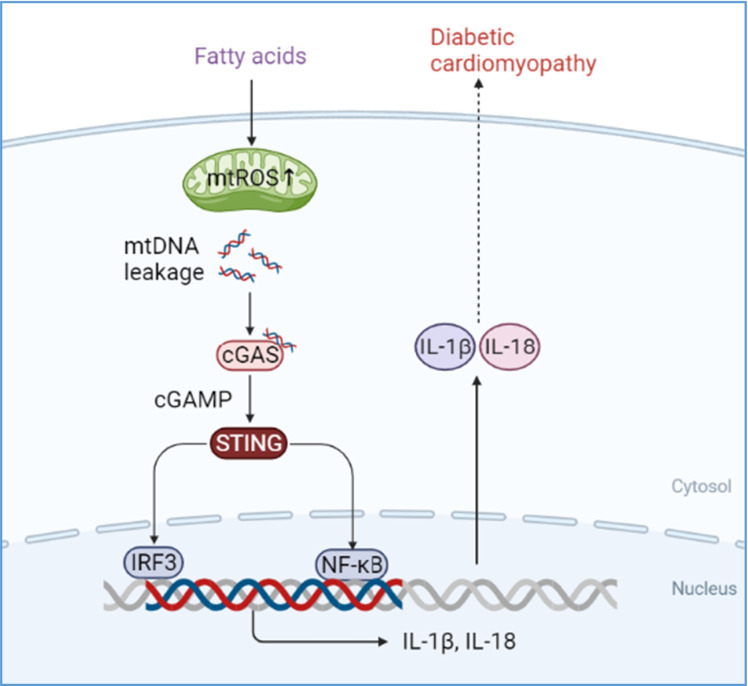

**Supplementary Information:**

The online version contains supplementary material available at 10.1007/s10565-021-09692-z.

## Highlights

- Mitochondria-derived cytosolic DNA acts as a critical linker between hyperlipidemia-induced mitochondrial dysfunction and pathogenesis of DCM.
- cGAS-STING pathway mediates the lipotoxicity-induced myocardial dysfunction through sensing released cytosolic mtDNA.
- STING was identified as a new potential therapeutic target for the treatment of DCM.

**Supplementary Information:**

The online version contains supplementary material available at 10.1007/s10565-021-09692-z.

## Introduction

The International Diabetes Federation estimates that by 2040, nearly 500 million people will be overweight and insulin resistant, and 642 million people will be affected by type 2 diabetes (T2D) (Ogurtsova et al. 2017). Chronic complications of T2D are the main hazards of diabetes, which often involve the heart, brain, kidney, and other vital organs. Among them, DCM is an important cause of heart failure in diabetic patients (Isfort et al. 2014; Seferović and Paulus 2015). T2D has many harmful effects on the heart, including lipid accumulation, abnormal energy metabolism, oxidative stress, inflammation, apoptosis, changes in fibrosis gene expression, and decreased left ventricular function (Peterson and Gropler 2020). In diabetic animal models, the increase of mitochondria-mediated cardiac apoptosis is a major event in DCM development (Bhagani et al. 2020; Cai et al. 2002).

As the energy metabolism center of sugar, fat, and protein, mitochondria account for nearly 30% of the volume of mature cardiomyocytes. Besides, mitochondria are also the places where intracellular signals integrate and regulate cell homeostasis (Mottis et al. 2019). Under stress conditions, damaged mitochondria can release some pro-inflammatory signals, such as reactive oxygen species (ROS), in response to changes in the intracellular environment (Chen and Zweier 2014; Dan Dunn et al. 2015). In some pathological conditions, such as auto-immune diseases and obesity, increased mitochondrial metabolic stress can lead to excessive ROS production and destruction of mitochondria, which triggers the release of mitochondrial DNA (mtDNA) into the cytoplasm (Bai et al. 2017; Ishikawa et al. 2009). In patients with T2D disease, the increase of myocardial triglyceride content is significantly related to the impairment of left ventricular diastolic function (Rijzewijk et al. 2008; Schulze et al. 2016). Previous studies have shown that DCM induced by fatty acids is associated with mitochondrial dysfunction, oxidative stress, and inflammation. However, the exact molecular mechanism of fatty acid-induced inflammation and cell death in DCM is still unclear.

cGMP-AMP (cGAMP) synthase (cGAS, also known as MB21D1) is considered to be a cytoplasmic DNA biosensor that recognizes DNA from pathogens (bacteria, viruses, etc.). It activates type I interferon response by synthesizing secondary messenger 2′3′-cGAMP in eukaryotic cells in response to the virus and microbial infection (Cheng et al. 2020; Ma and Damania 2016; Morehouse et al. 2020). cGAMP and its junction protein interferon gene stimulating protein (STING, also known as TMEM173) binding promote the translocation of STING from the endoplasmic reticulum to Golgi and form a complex with tank-binding kinase 1 (TBK1), which is transferred to the internal lysosome where TBK1 phosphorylates transcription factors, including interferon regulatory factor 3 (IRF3) and nuclear factor-kappa B (NF-κB), to initiate signal cascade activation of innate immunity-related genes, including type I interferon (IFN) (Ding et al. 2020; Lam et al. 2014; Tanaka and Chen 2012; Zhang et al. 2019). The activation of cGAS-STING protects cells from various pathogens and cancers by enhancing the immune response. However, recent studies have shown that in addition to DNA of microbial origin, the cGAS-STING pathway can also be activated by its cytoplasmic mtDNA (Liu et al. 2016; West and Shadel 2017).

Given that the mitochondrial metabolic stress can lead to mtDNA release and the cGAS-STING system can be activated by cytosolic mtDNA, it is worth noting whether impaired mitochondria contribute DCM through mtDNA-mediated activation of the cGAS-STING pathway. Here, we established an obesity-related DCM mouse model and observed the presence of cytosolic mtDNA, activation of cGAS/STING, and its downstream targets during DCM. Further analysis in palmitic acid (PA)-induced lipotoxic cell model showed PA-induced increase of cytosolic double-stranded DNA (dsDNA) and activation of cGAS/STING pathway in a dose-dependent manner. Knockdown of STING in PA-treated H9C2 cells and treatment with STING inhibitor in high-fat diet (HFD)-fed db/db mice can respectively block cell death and cardiac dysfunction. Our novel observations suggest that cytosolic mtDNA contributes to DCM through activation of cGAS/STING-mediated inflammatory pathway, indicating that functional inhibition of STING could be a potential therapeutic strategy for DCM patients.

## Methods

### Materials

Palmitic acid ( PA) and N-acetyl-l-cysteine (NAC) were obtained from Sigma (St. Louis, MO, USA). Mitochondria-targeted superoxide dismutase mimetic (mito-TEMPO) was purchased from Santa Cruz Biotechnology (Dallas, TX, USA). STING siRNA and the scrambled siRNA were acquired from RiboBio, Guangzhou, China. The fluorescein isothiocyanate (FITC) and cyanine dye 3 (Cy3) secondary antibodies used in immunofluorescence staining were purchased from Bioss, Beijing, China. In situ cell death detection kit was obtained from Roche, Switzerland. The whole gene DNA extraction kit was purchased from FOREGENE, Chengdu, China. The mitochondrial DNA extraction kit was purchased from BioVision, USA. Other chemicals in this study were of analytical grade.

### Cell culture and treatment

Cells were cultured as described previously (Zhao et al. 2016). Rat myocardial cells (H9C2) were subcloned from a cloned cell line of BD1X rat embryonic heart tissue, provided by the Institute of Myocardial Electrophysiology of Southwest Medical University in Luzhou, China. H9C2 cells were cultured in DMEM (Hyclone, USA) with 10% fetal bovine serum (Sciencell, USA), 100 IU ml^−1^ penicillin, and 100 μg ml^−1^ streptomycin (Beyotime, China) under 5% CO_2_ and ambient O_2_ at 37 °C (Thermo Scientific, USA).

### Animals

Male db/db and db/ + mice (4–5 weeks old) were got from Teng Xin, Chongqin, China. All mice were raised in a specific-pathogen-free (SPF) environment (humidity 50 ± 5%, temperature 20–22 °C). db/db were fed with a 60 kcal% fat diet (HFKbio, China) for 8–12 weeks to establish diabetic cardiomyopathy. The body weight of mice was measured every week, and fasting blood glucose was measured every 2 weeks. Mice treated with STING inhibitor were injected intraperitoneally with 750 nmol C-176 (Selleck, USA) per mouse daily in 200 μl corn oil (Selleck, USA) for 8 weeks. In this study, all animal experiment procedures were in accordance with the guidelines of the National Institutes of Health (NIH, Bethesda, USA) and Southwest Medical University (approval number: 201903–59).

### Echocardiography

Echocardiography was performed as described before (Wei et al. 2018). Briefly, echocardiograms were performed by a Vevo'3100 ultrasound (VisualSonics, Canada). Mice were anesthetized with 1.5–2% isoflurane before echocardiography. Cardiac function parameters were collected, including ejection fraction (EF), fractional shortening (FS), and peak E/A ratio.

### Serum triglycerides’ and inflammatory cytokines’ assays

Blood samples in each group were kept at room temperature for 30 min, and then centrifuged at 3000* g* for 15 min (4 °C). After then, the plasma samples were packed in Eppendorf tubes (EP tubes) and stored at − 80 °C for the subsequent analyses. The serum triglyceride level was determined by a rapid, convenient, and sensitive triglyceride detection kit (Nanjing Jiancheng Bioengineering Research Institute, China). The serum inflammatory cytokines IL-1β and IL-18 were detected using ELISA kits from Andy Gene, Beijing, China.

### Histological analysis

Histological changes were analyzed using hematoxylin–eosin (H&E) staining, TdT-mediated dUTP Nick-End Labeling (TUNEL) staining, and immunohistochemical staining as previously reported (Liu et al. 2017; Xiao et al. 2018). Simply, the tissue was fixed with 4% paraformaldehyde, and then dehydrated and paraffin-embedded. The hearts were cut into slices with a thickness of 4 μm and incubated overnight in a thermostat at 37 °C. Then, the slices were deparaffinized and rehydrated. After that, the morphology of cardiomyocytes was observed by H&E staining (Solebo). Besides, cardiomyocyte apoptosis was observed by TUNEL staining (Roche, Switzerland). Furthermore, myocardial fibrosis was evaluated by immunohistochemical staining. After being incubated with 3% H2O2 and 10% goat serum for 20 min and 1 h at room temperature respectively, the slices were incubated overnight with anti-CTGF (1:100, Santa Cruz, USA) and anti-COL1A1 (1:100, Santa Cruz, USA) at 4 °C and incubated with anti-mouse horseradish peroxidase reagent (37 °C, 1 h) and 3,3 N-diaminobenzidine tertrahydrochloride (DAB, room temperature, 5 min). Finally, the slices were observed with an optical microscope.

### DNA isolation and mtDNA analysis

The experiment was carried out as described previously (Bai et al. 2017). Briefly, the cultured cardiomyocytes and the freshly purified mouse cardiac tissue were divided into two equal volumes. Whole-cell genomic DNA was extracted by centrifugation column using a DNA extraction kit (FOREGENE, China). The other used a mitochondrial DNA (mtDNA) extraction kit (BioVision, USA) to extract and purify mtDNA. Cytoplasm free from nuclear, mitochondrial, and endoplasmic reticulum contamination was obtained by high-speed centrifugation. DNA was then isolated from these pure cytoplasmic components using a QIA Quick nucleotide removal column (QIAGEN, Germany). Quantitative PCR was performed using nuclear DNA primers (Tert) and mtDNA primers (Dloop1–3 and mtND4) for whole-cell extracts and cytoplasmic portions. The cycle threshold (CT) of mtDNA abundance in whole-cell extracts was used as normalized control, which effectively standardizes the sample and controls any change in the total amount of mtDNA in the sample.

### Western blot

Western blot analysis was performed as described before (Costantino et al. 2019). Total proteins in cells or tissues were lysed using RIPA buffer (Beyotime, China) and protein concentrations in cell lysates were determined using a bicinchoninic acid kit (BCA, Beyotime, China). The samples were separated by sodium salt-polyacrylamide gel electrophoresis (SDS-PAGE) and then transferred to 0.45-μm polyvinylidene fluoride (PVDF, Millipore, USA) membranes. After being blocked with 5% BSA for 1 h, membranes were incubated with primary antibodies including cGAS (1:1000, Santa Cruz, USA), STING (1:1000, CST, USA), p65 (1:500, CST, USA), p-p65 (1:1000, CST, USA), IRF3 (1:1000, Santa Cruz, USA), p-IRF3 (1:1000, CST, USA), IL-1β (1:1000, CST, USA), and Tubulin/GAPDH (1:5000, CST, USA) at 4 °C overnight. Then, the membranes were washed three times with TBST and incubated at room temperature with secondary antibodies for 1 h. Finally, the protein bands were visualized with an ECL luminescence reagent. Cytosolic proteins were normalized to Tubulin or GAPDH.

### Real-time PCR

Real-time PCR was performed as described before (Li et al. 2019a). Samples were homogenized in TRIzol (Invitrogen, USA), and total RNA was extracted from tissues or cells with an RNA extraction kit (TIANGEN, China). Then, 1 µl was taken for RNA OD value, OD260/280 and OD260/230, and the remaining RNA was reversely transcribed into cDNA and stored at − 80 °C for a long time (QIAGEN, Germany). Quantitative PCR reactions were performed using Applied Biosystems™ SYBR™ Green (Thermo Fisher Scientific, USA) and quantitated using the qTOWER 3G detection system (Germany). Duplicate runs of each sample were normalized to GAPDH to determine relative expression levels. Primer synthesis was carried out by Sangon Biotech, Shanghai, China. The primers used in this study are listed in supplementary table 1.

### Immunofluorescence staining

The experiment was carried out as described (Haag et al. 2018). Briefly, H9C2 were grown to a certain density on a 6-well plate overlay. Then, the cells were fixed in 4% paraformaldehyde and drilled with 0.2% Triton and blocked with 5% bovine serum albumin (BSA, Solarbio, China). Subsequently, the cells were incubated with the primary antibodies including mitofilin (1:100, Abcam, UK), dsDNA (1:100, Santa Cruz, USA), STING (1:100, CST, USA), GM130 (1:100, Santa Cruz, USA), and p65 (1:100, CST, USA) overnight at 4 C, and then incubated with FITC- or CY3-bound secondary antibodies for 1 h. 4,6-Diamidino-2-phenylindole (DAPI, Abcam, UK) was used for nuclear staining. Finally, cells were observed by a confocal microscope (Leica, Germany).

### Statistical analysis

Blots were converted to grayscale and densitometry analysis was performed in ImageJ. Co-localization analysis of immunofluorescence images was conducted using Mander’s overlap coefficient. Statistical analysis was performed by GraphPad Prism 6. For comparison between the two groups, an unpaired two-tailed *t* test was used. For multiple comparisons, one-way ANOVA was used with Turkey’s test No statistical method was used to predetermine sample size. Statistical significance was set at *P < 0.05, **P < 0.01, and ***P < 0.001.

## Results

### Diabetic cardiomyopathy occurred in HFD-fed db/db mice

**Fig. 1:**
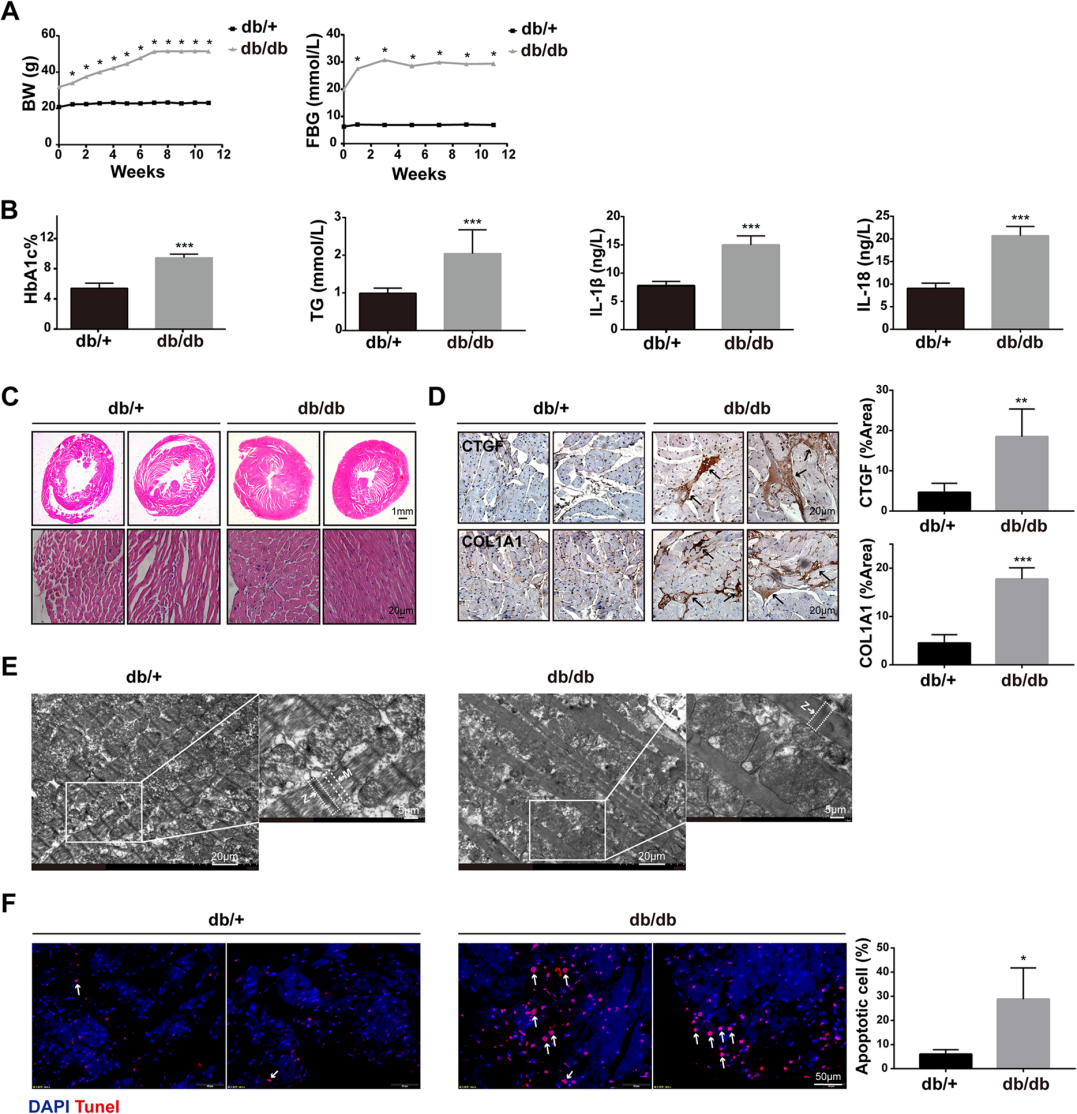
Diabetic cardiomyopathy occurred in HFD-fed db/db mice. **A** Dynamic changes of body weight (BW) and fasting blood glucose (FBG) in db/ + and db/db mice during ND and HFD feeding, respectively (*n* = 8, *P < 0.05 vs 0 week, using unpaired two-tailed *t* test). **B** The level of HbA1c, TG, IL-1β, and IL-18 in the blood of two groups of mice (*n* = 4, ***P < 0.001 vs db/ + group, using unpaired two-tailed *t* test). **C** Representative images of the morphological analysis by H&E staining of heart tissue. **D** CTGF and COL1A1 expressions in hearts of HFD-fed db/db mice were visualized by IHC staining (*n* = 4, **P < 0.01, ***P < 0.001 vs db/ + group, using unpaired two-tailed *t* test). **E** Representative transmission electron microscopy images of myofilament arrangement in db/ + mice and db/db mice (*n* = 4). The arrow indicated Z-line or M-line. **F** Representative images of cardiomyocyte apoptosis reflected by TUNEL staining, counterstained with DAPI (blue). The arrow indicated apoptotic cell (*n* = 4, *P < 0.05 vs db/ + group, using unpaired two-tailed *t* test)

Given that fatty acid (FA) oxidation accounts for 60 to 90% of mitochondrial ATP generation under normal conditions and FA accumulation is characteristic of the diabetic heart, we established obesity-related diabetic mouse model to induce diabetic cardiomyopathy by feeding db/db mice with HFD for 3 months. Our data showed that compared with the db/ + mice, the body weight (BW) and fasting blood glucose (FBG) of db/db mice fed with HFD were significantly higher (Fig. 1A), as well as hemoglobin A1c (HbA1c) and triglyceride (TG) (Fig. 1B). In addition, the detection of inflammatory markers showed that IL-1β and IL-18 in plasma of db/db increased (Fig. 1B). H&E staining showed that compared with db/ + , db/db fed with HFD showed obvious myocardial hypertrophy, narrowing of left ventricular cavity, myocardial fibrosis, and even breakage (Fig. 1C). Immunohistochemical staining showed that the CTGF- and COL1A1-labeled fibers in the myocardial interstitium of db/db were significantly increased, suggesting that HFD-induced myocardial fibrosis in db/db mice (Fig. 1D). An electron microscope showed that the myocardial myofilament bundles of db/ + mice were arranged neatly and the Z-line and M-line were clearly visible. By contrast, the myocardium of db/db mice was disordered or even broken, the Z- and M-lines were blurred (Fig. 1E). In addition, more apoptotic cells were observed by TUNEL staining in the myocardial interstitium of db/db fed with HFD (Fig. 1F). Together, our data indicate that diabetic cardiomyopathy occurred in HFD-fed db/db.

### Mitochondria were impaired with mtDNA release in cardiomyocytes from HFD-fed db/db mice

**Fig. 2:**
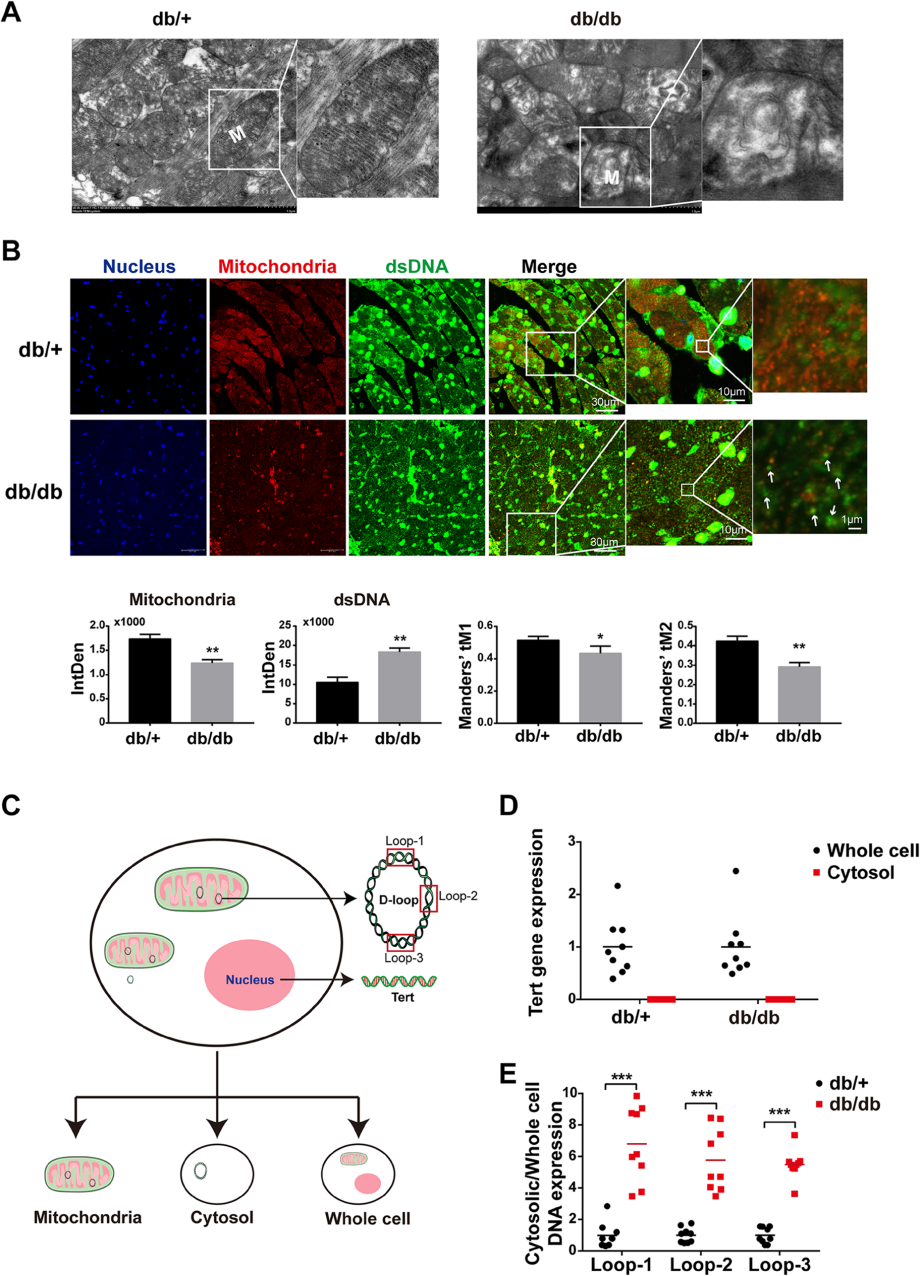
Mitochondria were impaired with mtDNA release in cardiomyocytes from HFD-fed db/db mice. **A** Representative transmission electron microscopy images of cardiomyocyte mitochondria in db/ + mice and db/db mice (*n* = 4). **B** Detection and quantification of dsDNA and mitofilin in cytoplasm of cardiomyocytes in two groups of mice by immunofluorescence double labeling, labeled mitochondria with mitofilin (red), labeled dsDNA with anti-dsDNA (green), and labeled nucleus with DAPI (blue). Co-localization analysis was done by Mander’s overlap coefficient for mitofilin with dsDNA (tM1) and dsNDA with mitofilin (tM2) (*n* = 4, *P < 0.05, **P < 0.005 vs db/ + group, using unpaired two-tailed *t* test, the arrow indicated free dsDNA). **C** Schematic diagram of extraction and detection of whole-cell DNA, mitochondrial DNA, and cytoplasmic free DNA. **D** Quantitative analysis of nuclear gene Tert expression in whole cell and cytoplasm of myocardial tissue in two groups of mice (*n* = 9). **E** Cytosolic mtDNA content in freshly purified cardiomyocytes of db/ + mice and db/db mice (*n* = 9, ***P < 0.001 vs db/ + group, using unpaired two-tailed *t* test)

As the cytosolic mtDNA derived from damaged mitochondria is a potential inflammatory mediator, we sought to identify the mitochondrial morphology and mtDNA release in DCM. We firstly performed electron microscopic analysis. In db/ + , the structure of myocardial mitochondria was complete, the shape was round or oval, and the mitochondrial cristae were complete, rich, and arranged in parallel, whereas in db/db, the arrangement of mitochondria was disordered, swollen, and irregular, and most of the cristae were broken, fused, exfoliated, or even myelinated, and some vacuoles could be seen (Fig. 2A). These data confirmed that the mitochondria in cardiomyocytes from DCM were severely impaired. Subsequently, we performed co-immunostaining of mitofilin, the inner membrane protein, and dsDNA to assess the mtDNA release. As expected, we found that compared with db/ + mice, the signals of mitofilin in cardiomyocytes of db/db mice were significantly decreased. Interestingly, we observed a significant increase in the number of free dsDNA in the cytoplasm of cardiomyocytes from db/db mice (Fig. 2B). To quantitatively characterize the spatial relationship between mitochondria and free dsDNA, we calculated Mander’s overlap coefficients (MOC). As shown in Fig. 2B, the values of tM1 and tM2 in the db/db group were lower than those in the db/ + group, suggesting that the co-localization of mitochondria and free DNA decreased. To quantify the mtDNA release amount, we separated mitochondria and cytosol from the whole cell for the qRT-PCR experiment. The primer Tert and primer Loop 1–3 were used to detect nuclear DNA and mitochondrial DNA, respectively (Fig. 2C). Our results showed that Tert was not detected in the isolated and purified myocardial cytoplasmic DNA (Fig. 2D), suggesting that the free dsDNA in the cytoplasm was not the nuclear source, and the cytoplasmic DNA extracted in this study was of high purity, and no obvious nucleolysis occurred. After that, we used primer Loop 1–3 to detect mitochondrial DNA in the isolated and purified cytoplasmic DNA. Consistently, the levels of free Loop1, Loop2, and Loop3 in the cytoplasm of db/db mice were significantly higher than those of db/ + mice (Fig. 2E), indicating that the free dsDNA in the cytoplasm was mainly derived from mitochondria. Taken together, mitochondria were impaired with mtDNA release in cardiomyocytes from HFD-fed db/db.

### The cGAS-STING-IRF3/NF-κB pathway was activated in hearts of HFD-fed db/db mice

**Fig. 3:**
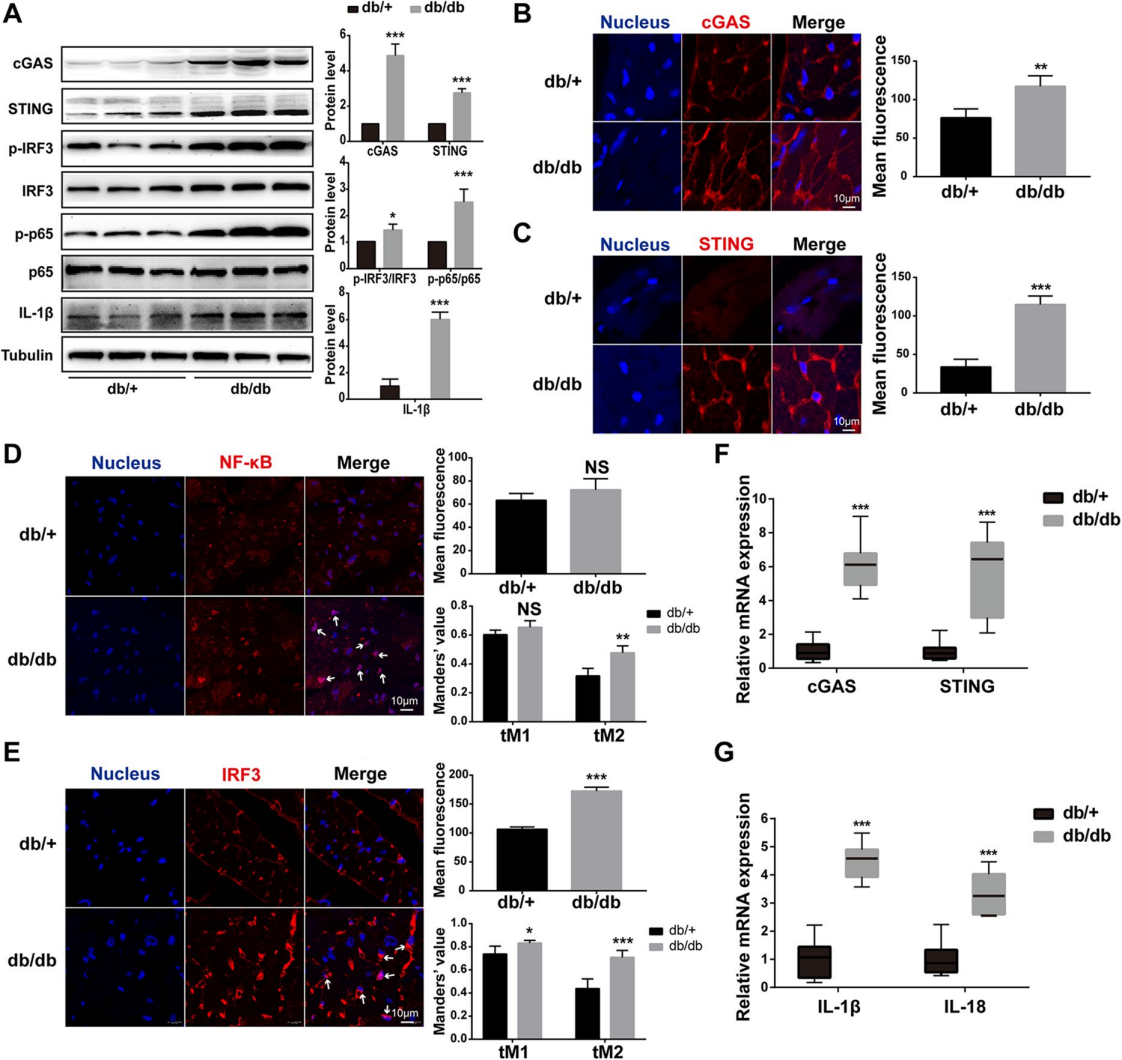
The cGAS-STING-IRF3/NF-κB pathway was activated in hearts of HFD-fed db/db mice. **A** The protein levels of cGAS, STING, p-IRF3/IRF3, p-p65/p65, and IL-1β in mouse myocardium of each group (*n* = 6, *P < 0.05, ***P < 0.001 vs db/ + group, using unpaired two-tailed *t* test). **B**–**E** Detection and quantification of cGAS, STING, NF-κB, and IRF3 in mouse heart of each group by immunofluorescence. The arrow indicated the activated NF-κB and IRF3. The Mander’s tM1 indicated that NF-κB or IRF3 co-localized with nucleus, whereas the Mander’s tM2 indicated nucleus that co-localized with NF-κB or IRF3 (*n* = 4, *P < 0.05, **P < 0.005, ***P < 0.001 vs db/ + group, using unpaired two-tailed *t* test). **F**, **G** Relative mRNA level of cGAS, STING, IL-1β, and IL-18 in mouse myocardium of each group (*n* = 6, ***P < 0.001 vs db/ + group, using unpaired two-tailed *t* test)

Given that mitochondrial damage led to mtDNA release into cytoplasm and cGAS is considered to be a cytoplasmic DNA biosensor, we next tested whether the cGAS-STING pathway was activated in hearts from HFD-fed diabetic mice. Expectedly, we found that the expression of cGAS and STING increased significantly in the cardiomyocytes of HFD-fed db/db mice (Fig. 3A). Immunofluorescence also showed that the expression of cGAS and STING was upregulated and clustered around the nucleus (Fig. 3B, C). In addition to activation of the cGAS and STING, the downstream targets, NF-κB and IRF3, were also activated in increased phosphorylated form (Fig. 3A, D, E), as well as the expression of NF-κB/IRF3-regulated IL-1β in the cardiomyocytes of HFD-fed db/db mice (Fig. 3A). Moreover, we found that the co-localization of NF-κB/IRF3 and nucleus increased by immunofluorescence, which further suggested their activation (Fig. 3D, E). Likewise, the increased mRNA levels of cGAS and STING in HFD-fed db/db mice were confirmed by RT-PCR (Fig. 3F), as well as the IL-1β and IL-18 (Fig. 3G). Taken together, these results suggested that the cGAS-STING-IRF3/NF-κB pathway was activated in hearts of HFD-fed db/db mice.

### PA-induced mitochondrial ROS led to mitochondrial damage and mtDNA release in H9C2 cells

**Fig. 4:**
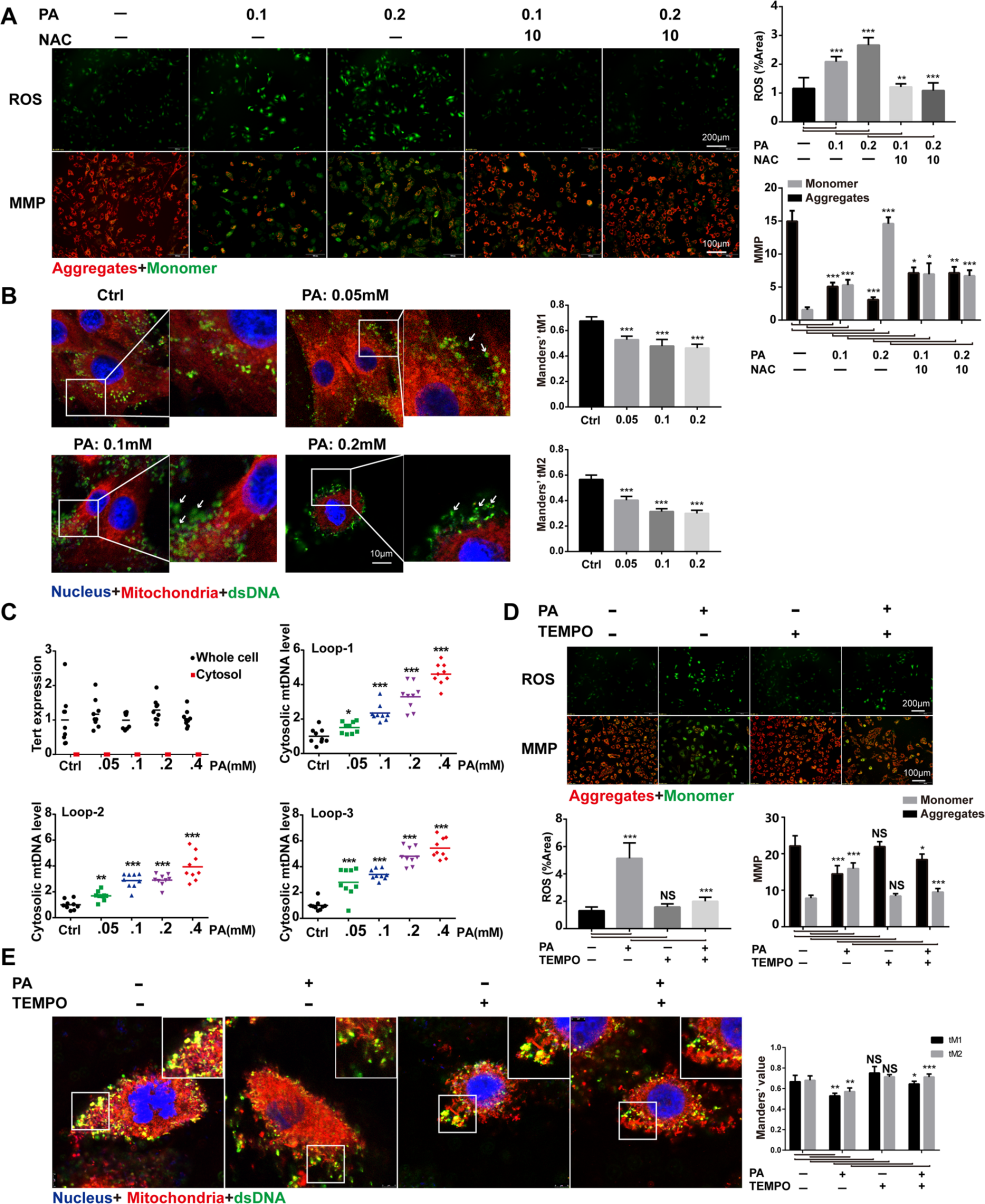
PA-induced mitochondrial ROS led to mitochondrial damage and mtDNA release in H9C2 cells. **A** ROS accumulation and the mitochondrial membrane potential (MMP) in H9C2 cells treated with PA for 24 h. ROS level was measured by DCFH-DA fluorescence and MMP was detected by JC-1 staining (*n* = 4, *P < 0.05, **P < 0.005, ***P < 0.001 vs indicated group, using one-way ANOVA followed by Turkey’s test). **B** Confocal fluorescence microscopic images of H9C2 cells after 24-h PA treatment, labeling dsDNA with anti-dsDNA (green), mitochondria with mito-tracker (red), and nuclei with DAPI (blue). The Mander’s tM1 indicated that mitochondria co-localized with the dsDNA, whereas the Mander’s tM2 indicated dsDNA that co-localized with mitochondria (*n* = 5, ***P < 0.001 vs ctrl group, using unpaired two-tailed *t* test). The arrow indicated free dsDNA. **C** Nuclear-encoded Tert gene expression in whole-cell and cytosolic extracts, and cytosolic mtDNA content from PA-treated H9C2 cells (*n* = 9, ***P < 0.001 vs ctrl group, using unpaired two-tailed *t* test). **D** ROS accumulation and MMP in PA-treated H9C2 cells (*n* = 4, PA: 0.2 mM for 2 h, mito-TEMPO: 0.1 mM for 2 h, *P < 0.05, ***P < 0.001 vs indicated group, using one-way ANOVA followed by Turkey’s test). **E** Confocal fluorescence microscopic images of H9C2 cells after PA treatment, labeling dsDNA with anti-dsDNA (green), mitochondria with anti-mitofilin (red), and nuclei with DAPI (blue). The Mander’s tM1 indicated that mitochondria co-localized with dsDNA, whereas the Mander’s tM2 indicated dsDNA that co-localized with mitochondria (*n* = 4, PA: 0.2 mM for 2 h, mito-TEMPO: 0.1 mM for 2 h, *P < 0.05, **P < 0.005, ***P < 0.001 vs indicated group, using one-way ANOVA followed by Turkey’s test)

To investigate whether the lipotoxicity mediates the activation of the cGAS-STING-IRF3/NF-κB pathway in hearts of HFD-fed db/db mice, we next used the H9C2 cell line treated by PA as a high fat-induced lipotoxic cell model. As shown in Fig. 4A, PA treatment led to an increase of ROS level and mitochondrial damage, which were both reversed by NAC, an inhibitor of ROS, indicating that PA-induced ROS led to mitochondrial damage. To confirm that PA treatment leads to mtDNA release into the cytoplasm, we performed co-immunostaining of mitochondria and dsDNA. As shown in Fig. 4B, PA induced an increase in cytoplasmic free dsDNA in a dose-dependent manner. Further study by qRT-PCR analysis revealed that the increased cytosolic dsDNA induced by PA was derived from mitochondria (Fig. 4C). To investigate the source of ROS in the process of mitochondrial injury induced by PA, we pre-treated H9C2 cells with mitochondrial-specific ROS scavenger mito-TEMPO. We found that mito-TEMPO could significantly reduce PA-induced intracellular ROS activation and improve mitochondrial membrane potential (Fig. 4D). In addition, we evaluated the leakage of mtDNA by fluorescence confocal analysis of dsDNA, mitochondria, and nucleus in PA-treated H9C2 cells. The results showed that mtDNA leakage in the cytoplasm of H9C2 cells treated with PA increased, while mito-TEMPO treatment of H9C2 cells in advance could significantly reduce mtDNA leakage induced by PA (Fig. 4E). In summary, these data showed that PA caused mitochondrial damage and mtDNA leakage mainly by activating mitochondrial ROS.

### PA-induced activation of the cGAS-STING pathway in H9C2 cells

**Fig. 5:**
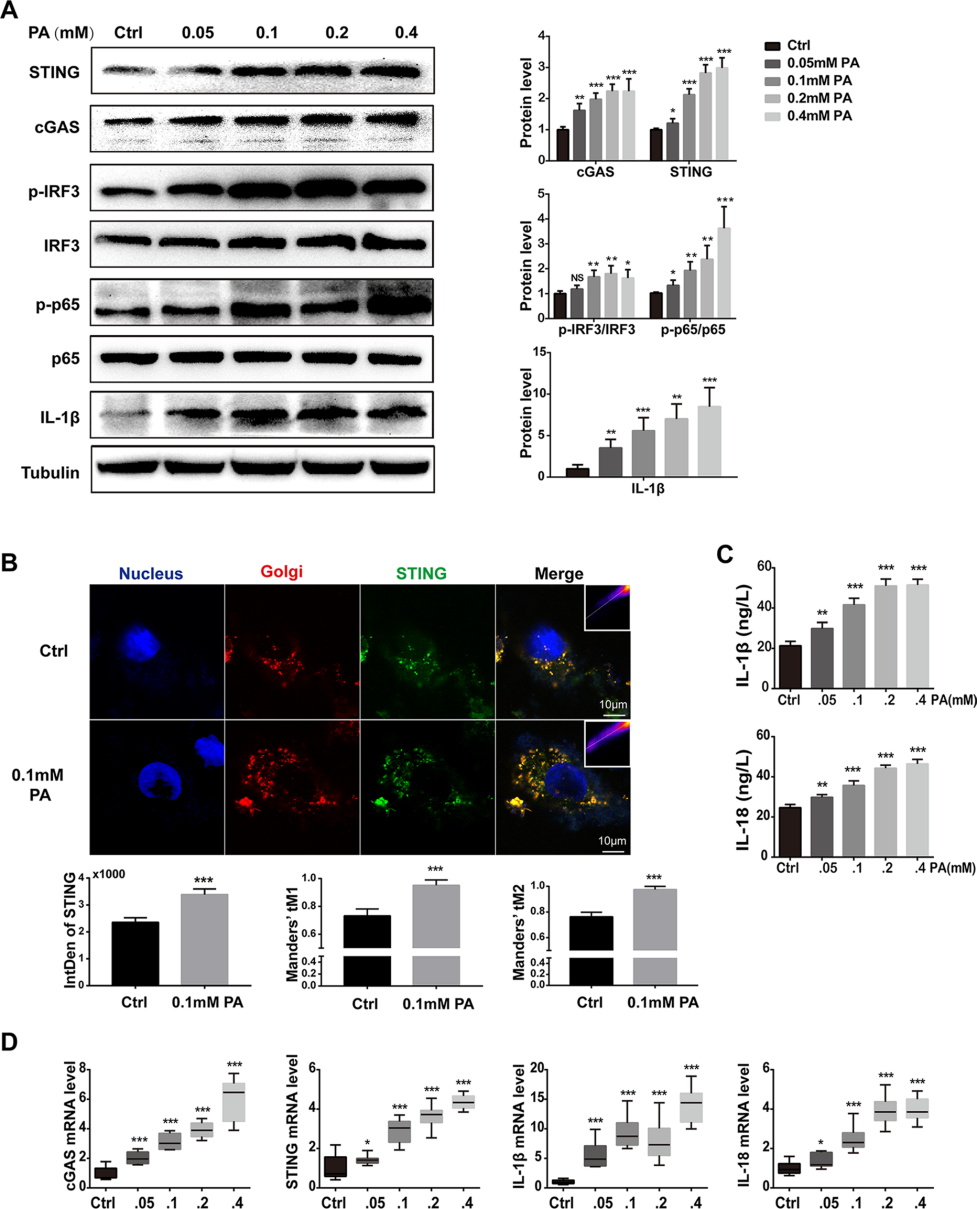
PA-induced activation of the cGAS-STING pathway in H9C2 cells. **A** The protein levels of cGAS, STING, p-IRF3/ IRF3, p-p65/p65, and IL-1β in H9C2 treated with PA (*n* = 6, *P < 0.05, **P < 0.01, ***P < 0.001 vs ctrl group, NS: no significance, using unpaired two-tailed *t* test). **B** Confocal fluorescence microscopic images of H9C2 cells after treatment with 0.1-mM PA for 24 h, labeling STING with anti-STING (green), Golgi with GM130 (red), and nuclei with DAPI (blue). Quantification of STING was detected by integrated density (IntDen). The Mander’s tM1 indicated that Golgi co-localized with STING, whereas the Mander’s tM2 indicated STING that co-localized with Golgi (*n* = 5, ***P < 0.001 vs ctrl group, using unpaired two-tailed *t* test). **C** The concentrations of IL-1β and IL-18 in the supernatant after H9C2 cells were stimulated by PA with concentration gradient for 24 h (*n* = 6, **P < 0.01, ***P < 0.001 vs ctrl group, using unpaired two-tailed *t* test). **D** Relative mRNA level of cGAS, STING, and inflammatory genes IL-1β and IL-18 in H9C2 cells treated with PA for 24 h (*n* = 6, *P < 0.05, ***P < 0.001 vs ctrl group, using unpaired two-tailed *t* test)

To elucidate the effect of PA-induced mtDNA release, we next evaluated the activation of the cGAS-STING in PA-treated H9C2. As shown in Fig. 5A, PA treatment led to an elevated protein level of cGAS and STING in a dose-dependent manner in H9C2 cells. In addition, the downstream targets, phosphorylated IRF3 and NF-κB, were also activated by PA treatment in a dose-dependent manner, as well as IL-1β, which was regulated by IRF3 and NF-κB (Fig. 5A). Given that the function of STING is determined not only by its content but also by its location. We next performed co-immunostaining of STING and Golgi matrix protein 130 (GM130), a Golgi marker. In H9C2 cells without PA treatment, STING was weakly co-localized with GM130, while PA treatment induced strong co-localization, which directly indicated the functional activation of STING (Fig. 5B). Consistently, IL-1β and IL-18 in the supernatant of H9C2 cells after PA treatment were also increased in a dose-dependent manner (Fig. 5C), as well as the mRNA levels of cGAS, STING, IL-1β, and IL-18 (Fig. 5D). Taken together, these results indicated that activation of the cGAS-STING pathway is involved in PA-induced myocardial inflammation.

### Extracted mtDNA is sufficient to activate cGAS-STING signaling in H9C2 cells

**Fig. 6:**
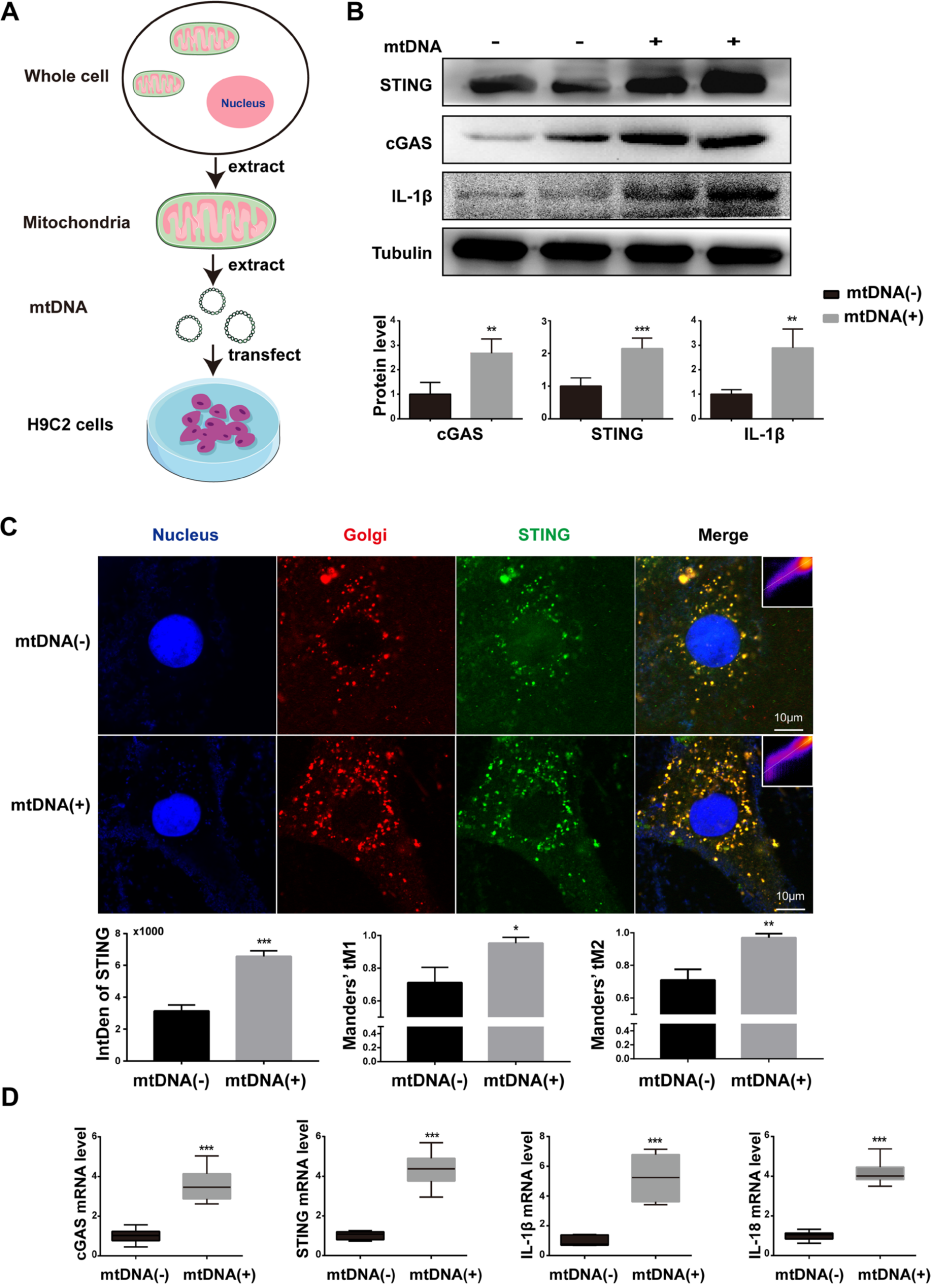
Extracted mtDNA is sufficient to activate cGAS-STING signaling in H9C2 cells. **A** Flow chart of mitochondrial DNA extraction and transfection.** B** The protein levels of cGAS, STING, and IL-1β after mtDNA transfection of H9C2 cells (*n* = 6, mtDNA 3 μg for 24 h, **P < 0.01, ***P < 0.001 vs mtDNA(-) group, using unpaired two-tailed *t* test). **C** Confocal fluorescence microscopic images of H9C2 cells after transfected with 3 μg mtDNA for 24 h, labeling STING with anti-STING (green), Golgi with GM130 (red), and nuclei with DAPI (blue). Quantification of STING was detected by integrated density (IntDen). The Mander’s tM1 indicated that Golgi co-localized with STING, whereas the Mander’s tM2 indicated STING that co-localized with Golgi (*n* = 4, *P < 0.05, **P < 0.01, ***P < 0.001 vs mtDNA(-) group, using unpaired two-tailed *t* test). **D** Relative mRNA level of cGAS, STING, and inflammatory genes IL-1β and IL-18 after mtDNA transfection of H9C2 cells (*n* = 6, ***P < 0.001 vs mtDNA(-) group, using unpaired two-tailed *t* test)

As the cGAS is not the mtDNA-specific DNA sensor, other sorts of DNA can also activate it. To confirm that mitochondria-derived mtDNA can activate the cGAS-STING pathway, we isolated and purified mtDNA to transfect into H9C2 cells. Then, the activation of the cGAS-STING pathway and downstream inflammatory activation level were detected by western blot and qRT-PCR. As shown in Fig. 6 A and C, cGAS and STING expression was activated after mtDNA transfection, accompanying the increased expression of IL-1β and IL-18. In addition, we performed co-immunostaining of STING and Golgi in PA-treated H9C2 cells to evaluate the activation of STING. The results indicated that STING aggregation to Golgi was significantly increased in mtDNA-transfected H9C2 cells (Fig. 6B), suggesting that STING was functionally activated by mtDNA treatment. In summary, these data showed that in PA-induced myocardial inflammation, the released cytoplasmic mtDNA acted as the ligand of the cGAS-STING system.

### Knockdown of STING blocked the PA-induced inflammation and apoptosis in H9C2 cells

**Fig. 7:**
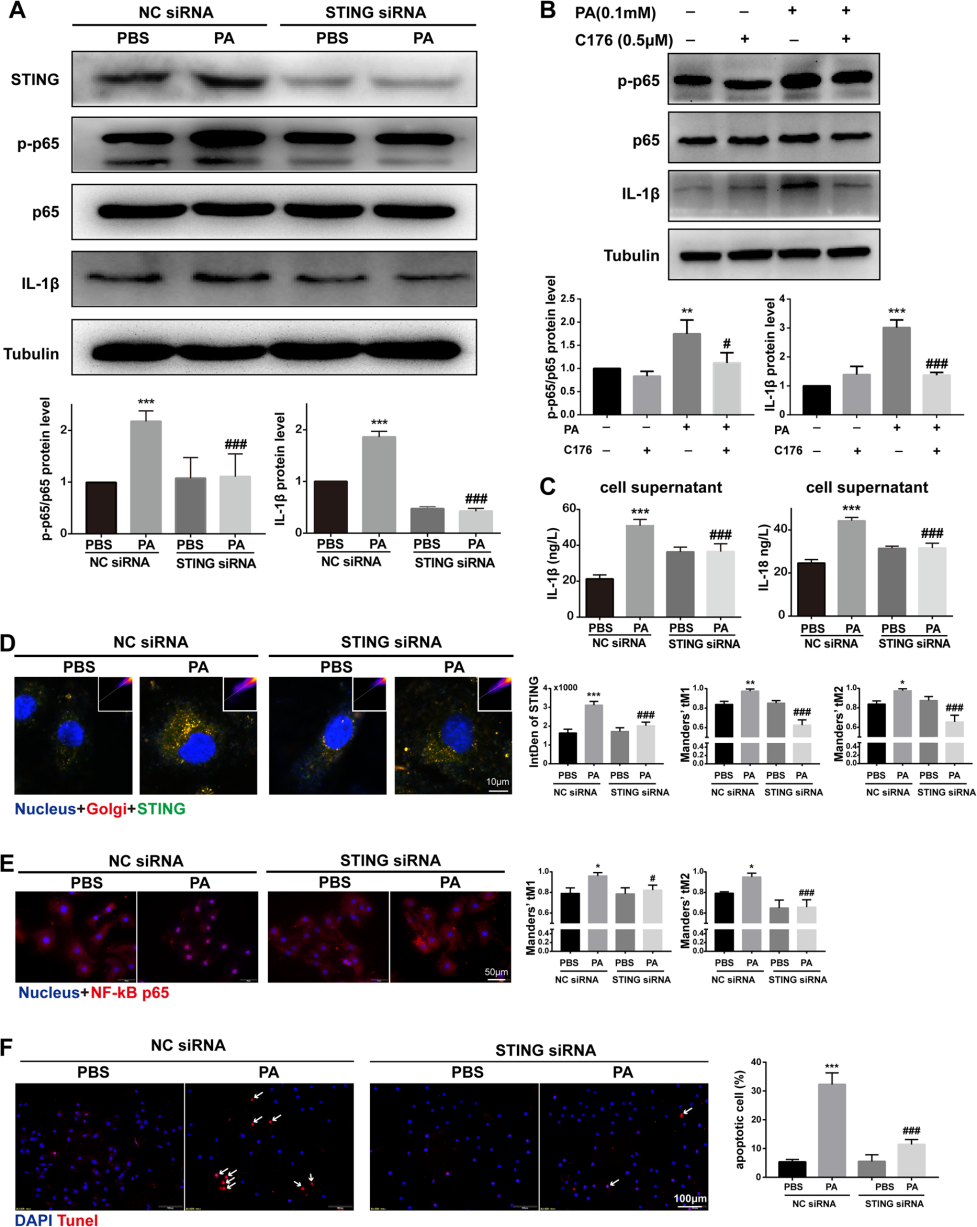
Knockdown of STING blocked the PA-induced inflammation and apoptosis in H9C2 cells. **A** The protein levels of STING, p-p65/p65, and IL-1β in PA-treated H9C2 cells after STING knockdown by siRNA (*n* = 6, ***P < 0.001 vs PBS group with NC siRNA; ^###^*P* < 0.001 vs PA group with NC siRNA, using one-way ANOVA followed by Turkey’s test). **B** The protein levels of p-p65/p65 and IL-1β in PA-treated H9C2 cells pre-treated with STING inhibitor C176 (*n* = 4, **P < 0.01, ***P < 0.001 vs ctrl group; ^#^*P* < 0.05, ^###^*P* < 0.001 vs PA group, using one-way ANOVA followed by Turkey’s test). **C** The concentration of IL-1β and IL-18 in the supernatant of H9C2 cells transfected by STING siRNA (*n* = 6, ***P < 0.001 vs PBS group with NC siRNA; ^###^*P* < 0.001 vs PA group with NC siRNA, using one-way ANOVA followed by Turkey’s test). **D** Confocal fluorescence microscopic images of PA-treated H9C2 cells after STING knockdown by siRNA, labeling STING with anti-STING (green), Golgi with GM130 (red), and nuclei with DAPI (blue). Quantification of STING was detected by integrated density (IntDen). The Mander’s tM1 indicated that Golgi co-localized with STING, whereas the Mander’s tM2 indicated STING that co-localized with Golgi (*n* = 4, *P < 0.05, **P < 0.01, ***P < 0.001 vs PBS group with NC siRNA; ^###^*P* < 0.001 vs PA group with NC siRNA, using one-way ANOVA followed by Turkey’s test). **E** Representative images of immunofluorescence of NF-κB in H9C2 cells transfected by STING siRNA. The Mander’s tM1 indicated that NF-κB co-localized with nucleus, whereas the Mander’s tM2 indicated nucleus that co-localized with NF-κB (*n* = 4, *P < 0.05 vs PBS group with NC siRNA; ^#^*P* < 0.05, ^###^*P* < 0.001 vs PA group with NC siRNA, using one-way ANOVA followed by Turkey’s test). **F** Representative apoptosis images of H9C2 cells treated by PA after NC siRNA or STING siRNA transfected, reflected by TUNEL staining, counterstained with DAPI (blue). The arrow indicated apoptotic cell (*n* = 6, ***P < 0.001 vs PBS group with NC siRNA; ^###^*P* < 0.001 vs PA group with NC siRNA, using one-way ANOVA followed by Turkey’s test)

Given that STING functions as an effector in the cGAS-STING system, we sought to identify whether the inhibition of STING can reverse the effect of PA treatment in H9C2 cells. We employed siRNA to knock down the STING mRNA. In H9C2 cells transfected with STING siRNA, the expression of STING protein was significantly decreased (Fig. 7A, D), and the localization of STING in Golgi was significantly decreased (Fig. 7D), indicating that the transfection of STING siRNA was effective. As expected, STING knockdown could significantly inhibit the activation of NF-κB and the increase of IL-1β in H9C2 cells treated by PA for 24 h (Fig. 7A, E). Meanwhile, we have pre-treated H9C2 cells with C-176 (a small molecule inhibitor of palmitoylation of STING) and quantify IL-1β and p-p65 levels. The results showed that C176 could reduce the expression of IL-1β and phosphorylation of p65 induced by palmitic acid, which was similar to that of STING siRNA (Fig. 7B). In addition, STING knockdown could also significantly block the elevated secretion of IL-1β and IL-18 induced by PA treatment in the supernatant of H9C2 cells (Fig. 7C). Moreover, we also observed a significant anti-apoptotic effect of STING knockdown on PA-treated H9C2 cells (Fig. 7F). Taken together, these data directly indicated that knockdown of STING blocked the PA-induced inflammation and apoptosis in H9C2 cells.

### Inhibition of STING-ameliorated diabetic cardiomyopathy in HFD-fed db/db mice

**Fig. 8:**
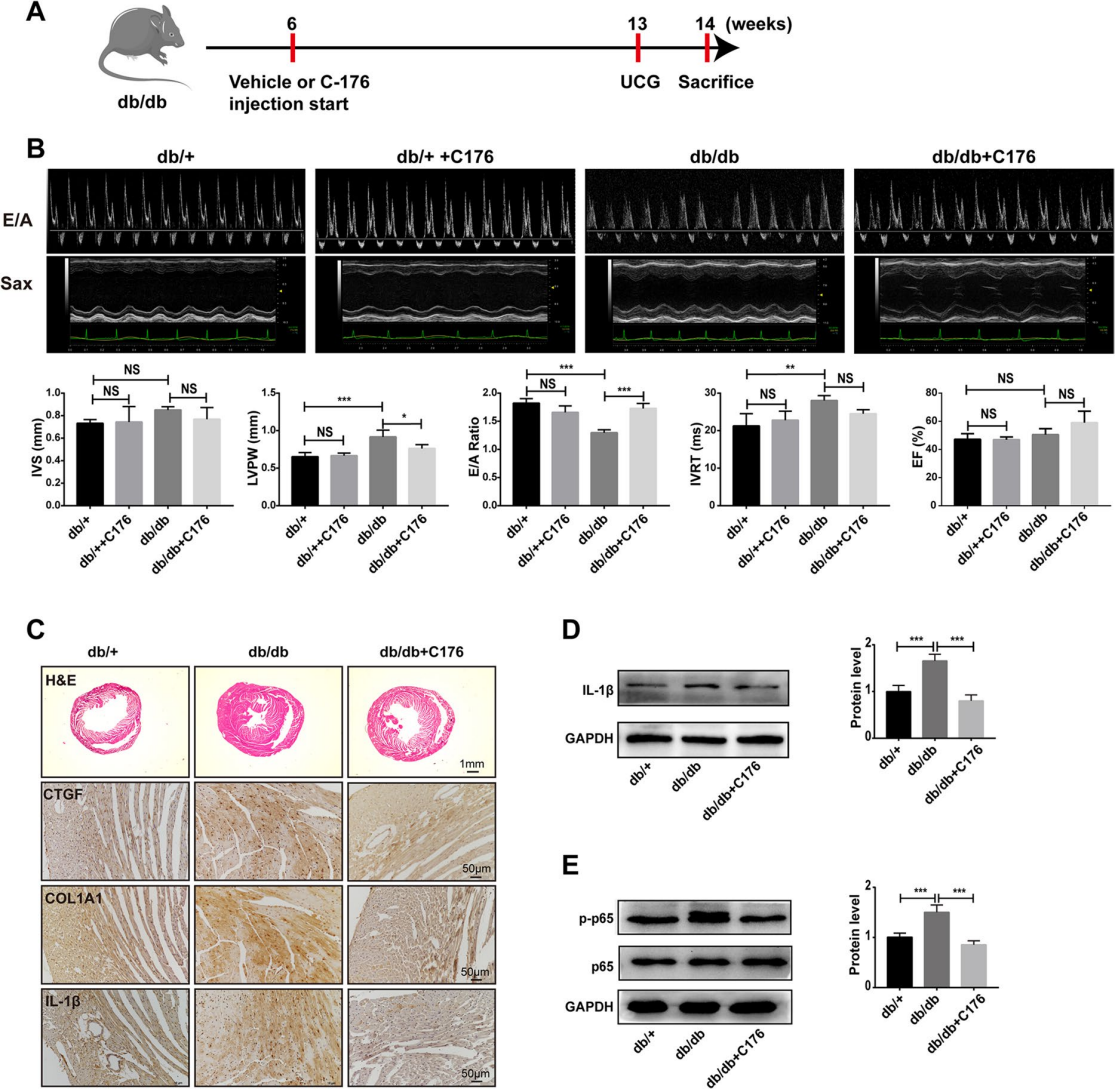
Inhibition of STING-ameliorated diabetic cardiomyopathy in HFD-fed db/db mice. **A** Flow chart of mouse feeding (C176, STING inhibitor, 750 nmol per mouse daily in 200 μl corn oil, intraperitoneal injection. UCG, ultrasound cardiogram). **B** Representative echocardiographic images of each group. IVS (interventricular septal thickness), LVPW (posterior wall thickness of left ventricle), EF% (ejection fraction), E/A ratio and IVRT (isovolumic relaxation time) (*n* = 4, *P < 0.05, **P < 0.01, ***P < 0.001 vs indicated group, NS: no significance, using one-way ANOVA followed by Turkey’s test). **C** Representative mouse myocardial images of the morphological analysis by H&E staining and fibrosis analysis labeled with CTGF, and COL1A1 by immunohistochemistry staining (*n* = 4). **D** The protein levels of IL-1β in mouse myocardium (*n* = 4, ***P < 0.001 vs indicated group, using one-way ANOVA followed by Turkey’s test). **E** The protein levels of p-p65/p65 in mouse myocardium (*n* = 4, ***P < 0.001 vs indicated group, using one-way ANOVA followed by Turkey’s test)

Since that knockdown of STING blocked the PA-induced inflammation and apoptosis in H9C2 cells, we supposed STING as a potential therapeutic target of DCM. To this end, we used C176, a specific inhibitor of STING, to intraperitoneally inject into HFD-fed db/db mice (Fig. 8A). As the body weight (BW) of db/db mice was stable from the 7 weeks of HFD (Fig. 1A), we intended to start the UCG at the 7 weeks of HFD and perform once a week. Unexpectedly, C176-treated db/db mice exhibited improved cardiac parameters than vehicle-treated db/db at the 7 weeks of HFD. As shown in Fig. 8B, C176 had a slight effect on cardiac diastolic function in db/ + mice, but without statistical significance. However, inhibition of STING can reverse the cardiac dysfunction in db/db mice fed with HFD, showing an increase in E/A ratio and a shortening of isovolumic relaxation time (IVRT), suggesting an improvement in diastolic cardiac function. In addition, inhibition of STING could partially improve myocardial hypertrophy induced by HFD but had no significant effect on myocardial contractile function (Fig. 8B). To further study the pathological changes, we performed H&E staining to observe the cardiac hypertrophy and immunohistochemistry to observe the myocardial fibrosis. The results showed that HFD feeding induced ventricular hypertrophy and myocardial fibrosis in db/db mice, which could be partially reversed by C176 treatment (Fig. 8C). Also, HFD feeding induced the increase of inflammatory cytokine IL-1β in db/db mice, while C176 treatment reduced the production of IL-1β (Fig. 8C), which was also confirmed by western blot (Fig. 8D). Besides, C176 treatment also blocked the HFD feeding-induced activation of NF-κB in db/db mice by inhibition of phosphorylated P65 (Fig. 8E). In a word, these results suggest that STING functions as a potential therapeutic target for diabetic cardiomyopathy.

## Discussion

As DCM is an important cause of heart failure in diabetic patients (Isfort et al. 2014; Seferović and Paulus 2015), it is critical to identify therapeutic targets to prevent disease progression. Recently, a growing body of evidence has demonstrated that the cGAS-STING system plays a central role in numerous diseases such as obesity, nonalcoholic fatty liver disease (NAFLD), and acute kidney injury (Bai et al. 2017; Luo et al. 2018; Maekawa et al. 2019). In this study, we observed the presence of mitochondrial damage, cytosolic mtDNA, and activation of the cGAS-STING signaling pathway in cardiomyocytes from an obesity-related DCM mouse model. Using a PA-induced lipotoxicity cell model, we determined that PA-induced mtROS overproduction resulted in mtDNA release, which subsequently activated the cGAS/STING signaling pathway and its downstream targets, NF-κB and IRF3. The activated NF-κB/IRF3 finally promoted the expression of inflammatory factors, IL-18 and IL-1β. Notably, either downregulation of STING in H9C2 cells or STING inhibitor injection to HFD-fed db/db mice could block the lipotoxicity-induced inflammation and cell death. These findings suggest that STING is a novel, critical molecule involved in the progression of DCM

In mature cardiomyocytes, mitochondria account for nearly 30% of the volume. It is well known that mitochondrial dysfunction plays a vital role in the pathological process of diabetic cardiomyopathy (López-Armada et al. 2013). A clinical study has reported that mitochondria in cardiomyocytes of diabetic patients showed fragmentation (Montaigne et al. 2014). Mitofilin, an essential protein involved in mitochondrial inner crest formation, was reported to be downregulated in the diabetic heart by proteome analysis and transgenic overexpression of mitofilin attenuated diabetes mellitus-associated cardiac and mitochondrial dysfunction (Thapa et al. 2015). However, the molecular mechanism linking mitochondrial dysfunction and cardiac cell death and inflammation is unclear. Here, we also visually observed the damage of the inner mitochondrial membrane of cardiomyocytes in diabetic mice through electron microscopy (Fig. 2). Of note, we found decreased mitofilin and increased cytoplasmic mtDNA in H9C2 cells treated with palmitic acid and myocardial tissue of HFD-fed db/db mice (Figs. 2 and 4), suggesting that mitochondrial damage characterized by mitofilin decreased resulted in mtDNA leakage into the cytosol. In addition, the DNA sensor system, cGAS-STING signaling, was activated in PA-treated H9C2 cells and diabetic hearts (Figs. 3 and 5). The extracted mtDNA treatment alone was sufficient to activate cGAS-STING and the downstream targets in vitro (Fig. 6). Together, these results suggested that mitochondria-derived cytosolic DNA acts as a critical linker between mitochondrial dysfunction and the pathogenesis of DCM.

It is widely recognized that excessive mitochondrial ROS causes mitochondrial dysfunction and induces cellular dysfunction in cardiomyocytes by compromising ATP production (Fauconnier et al. 2007; Zorov et al. 2014). On the one hand, previous studies have shown that myocardial cells in T1D and T2D animal models and diabetic patients generally show increased ROS, as well as mitochondrial morphological changes, mainly including mitochondrial fragmentation, crest fracture, and swelling (Galloway and Yoon 2015; Jarosz et al. 2017). On the other hand, hyperglycemia-induced mitochondrial fragmentation can be reversed by stimulating the antioxidant superoxide dismutase (SOD), suggesting a causal relationship between ROS and mitochondrial dysfunction, and controlling mtROS levels may be a strategy for treating DCM (Schilling 2015; Westermeier et al. 2015). However, the role of hyperlipidemia-induced mtROS in the progression of DCM is unknown. In this study, we observed excessive production of ROS and impaired mitochondria in H9C2 cells treated by palmitic acid in a dose-dependent manner (Fig. 4A). Although incubation with ROS scavenger NAC could effectively reduce the formation of ROS and reverse the mitochondrial function (Fig. 4A), it is not sure whether the overproduction of ROS is derived from mitochondria as the ROS is not only produced by mitochondria. Notably, we further observed that the mitochondria-targeted antioxidant, mito-TEMPO, significantly inhibited the PA-induced myocardial ROS production (Fig. 4D), indicating the overproduction of mtROS in cardiomyocytes under hyperlipidemia. Consistently, a recently published study reported that injection of mito-TEMPO for 30 days reduced cardiomyocyte apoptosis and improved cardiac hypertrophy and dysfunction in diabetic mice (Ni et al. 2016). Furthermore, we discovered that mito-TEMPO could not only block the mtROS overproduction but also significantly reduce the leakage of mitochondrial DNA, suggesting a mechanism of mtROS-induced cytosolic DNA increase (Fig. 4E). Taken together, our data revealed an early regulated axis of lipid/mtROS/mtDNA in obesity-related DCM.

As we have known, mtDNA is thought to be similar to bacterial DNA and contains pro-inflammatory, unmethylated CpG motifs (Collins et al. 2004). Previous studies have shown that escaping mtDNA can inflame the heart and even cause heart failure (Konstantinidis and Kitsis 2012; Oka et al. 2012). In a normal physiological state, escaped mtDNA and damaged mitochondria can be digested and degraded by lysosome-mediated autophagy and mitophagy, whereas in a variety of disease states, such as blood pressure overload and ischemia–reperfusion injury, excess mtDNA accumulates and activates the Toll-like receptor 9 (TLR9), resulting in persistently activated inflammation response (Oka et al. 2012). In addition, it has been shown that oxidized mitochondrial DNA could directly activate pyrin domain-containing protein 3 (NLRP3) during apoptosis (Shimada et al. 2012). Here, we report activation of the cGAS-STING system, accompanied by increased cytoplasmic mtDNA, in HFD-fed db/db and PA-treated H9C2 (Figs. 2, 3, 4, and 5). Moreover, the cGAS-STING pathway could be activated by the extracted mtDNA treatment only in cultured H9C2 cells (Fig. 6). Our study identified cGAS-STING, not TLR9 receptor, as another mtDNA sensor to mediate lipotoxicity-induced myocardial dysfunction.

As a DNA sensor system, the cGAS-STING pathway was first discovered as a mediator of type I IFN inflammatory responses in immune cells to defend against viral and bacterial infections (Ma and Damania 2016; Marinho et al. 2017). A growing body of evidence has shown that the cGAS-STING pathway was also activated by host DNA, which aberrantly localized in the cytosol, contributing to increased sterile inflammation, insulin resistance, and the development of NAFLD (Isfort et al. 2014; Luo et al. 2018). Following the activation of STING signaling, TBK1 is recruited and activated via its phosphorylated C-terminal tail (CTT) (Zhang et al. 2019). The activated TBK1 acts as a scaffold to recruit IRF3, which is then phosphorylated in a TBK1-dependent manner. The phosphorylated IRF3 enters the nucleus and promotes the expression of target genes such as interferon (Li et al. 2019b; Tanaka and Chen 2012). On the other hand, TBK1 also plays a role as the activator of NF-κB, which could promote not only interferon expression but also a transcription of pro-inflammatory and chemokine factors (Abe and Barber 2014). Consistently, our results demonstrated that activation of the cGAS-STING pathway was accompanied by increases of the downstream mediators, IRF3 and p65 (one form of NF-κB), and the downstream inflammatory factors, IL-18 and IL-1β (Figs. 3 and 5). Of note, both knockdowns of STING in PA-treated H9C2 cells (Fig. 7) and inhibition of STING with C176 injection (Fig. 8) can remarkably ameliorate myocardial inflammation and apoptosis. These data suggest cGAS-STING/IRF3/NF-κB axis acts as a mediator in the progression of DCM.

## Conclusion

Our study demonstrated that lipotoxicity-induced mtDNA release led to cardiac cell death and fibrosis by activation of cGAS-STING signaling and subsequent inflammation in the obesity-related DCM mouse model. These findings underline the significance of cGAS/STING signaling as a potential therapeutic target in DCM, and the preclinical efficacy of STING inhibition as a new therapeutic strategy for the treatment of DCM.

## Supplementary Information

Below is the link to the electronic supplementary material.

Supplementary file1 (DOCX 13 KB)

## Data Availability

The datasets generated during and/or analyzed during the current study are available from the corresponding author on reasonable request.

## Abbreviations

T2D: Type 2 diabetes
DCM: Diabetic cardiomyopathy
ROS: Reactive oxygen species
mtDNA: Mitochondrial DNA
cGAS: CGMP-AMP (cGAMP) synthase
STING: Interferon gene stimulating protein
TBK1: Tank-binding kinase 1
IRF3: Interferon regulatory factor 3
NF-κB: Nuclear factor-kappa B
IFN: Interferon
dsDNA: Double-stranded DNA
HFD: High-fat diet
PA: Palmitic acid
NAC: N-acetyl-l-cysteine
mito-TEMPO: Mitochondria-targeted superoxide dismutase mimetic
SPF: Specific-pathogen-free
NIH: National Institutes of Health
EF: Ejection fraction
FS: Fractional shortening
IVS: Interventricular septum
LVPW: Left ventricular posterior wall
EP tubes: Eppendorf tubes
H&E: Hematoxylin-eosin
TUNEL: TdT-mediated dUTP Nick-End Labeling
DAB: 3,3 N-diaminobenzidine tetrahydrochloride
CT: Cycle threshold
SDS-PAGE: Sodium dodecyl sulfate–polyacrylamide gel electrophoresis
PVDF: Polyvinylidene fluoride
BW: Body weight
FBG: Fasting blood glucose
HbA1c: Hemoglobin A1c
TG: Triglyceride
MOC: Mander’s overlap coefficients
FA: Fatty acid
GM130: Golgi matrix protein 130
UCG: Ultrasound cardiogram
siRNA: Small interfering RNA
SOD: Superoxide dismutase
TLR9: Toll-like receptor 9
NLRP3: Pyrin domain-containing protein 3
NAFLD: Nonalcoholic fatty liver disease
CTT: C-terminal tail

## References

[CR1] Abe T, Barber GN (2014). Cytosolic-DNA-mediated, STING-dependent proinflammatory gene induction necessitates canonical NF-κB activation through TBK1. J Virol.

[CR2] Bai J, Cervantes C, Liu J, He S, Zhou H, Zhang B, Cai H, Yin D, Hu D, Li Z, Chen H, Gao X, Wang F, O'Connor JC, Xu Y, Liu M, Dong LQ, Liu F (2017). DsbA-L prevents obesity-induced inflammation and insulin resistance by suppressing the mtDNA release-activated cGAS-cGAMP-STING pathway. Proc Natl Acad Sci U S A.

[CR3] Bai J, Cervantes C, He S, He J, Plasko GR, Wen J, Li Z, Yin D, Zhang C, Liu M, Dong LQ, Liu F (2020). Mitochondrial stress-activated cGAS-STING pathway inhibits thermogenic program and contributes to overnutrition-induced obesity in mice. Commun Biol.

[CR4] Bhagani H, Nasser SA, Dakroub A, El-Yazbi AF, Eid AA, Kobeissy F, Pintus G, Eid AH (2020). The mitochondria: a target of polyphenols in the treatment of diabetic cardiomyopathy. Int J Mol Sci.

[CR5] Cai L, Li W, Wang G, Guo L, Jiang Y, Kang YJ (2002). Hyperglycemia-induced apoptosis in mouse myocardium: mitochondrial cytochrome C-mediated caspase-3 activation pathway. Diabetes.

[CR6] Chen YR, Zweier JL (2014). Cardiac mitochondria and reactive oxygen species generation. Circ Res.

[CR7] Cheng Z, Dai T, He X, Zhang Z, Xie F, Wang S, Zhang L, Zhou F (2020). The interactions between cGAS-STING pathway and pathogens. Signal Transduct Target Ther.

[CR8] Collins LV, Hajizadeh S, Holme E, Jonsson IM, Tarkowski A (2004). Endogenously oxidized mitochondrial DNA induces in vivo and in vitro inflammatory responses. J Leukoc Biol.

[CR9] Costantino S, Akhmedov A, Melina G, Mohammed SA, Othman A, Ambrosini S, Wijnen WJ, Sada L, Ciavarella GM, Liberale L, Tanner FC, Matter CM, Hornemann T, Volpe M, Mechta-Grigoriou F, Camici GG, Sinatra R, Lüscher TF, Paneni F (2019). Obesity-induced activation of JunD promotes myocardial lipid accumulation and metabolic cardiomyopathy. Eur Heart J.

[CR10] Ding C, Song Z, Shen A, Chen T, Zhang A (2020). Small molecules targeting the innate immune cGAS-STING-TBK1 signaling pathway. Acta Pharm Sin B.

[CR11] Dan Dunn J, Alvarez LA, Zhang X, Soldati T (2015). Reactive oxygen species and mitochondria: a nexus of cellular homeostasis. Redox Biol.

[CR12] Fauconnier J, Andersson DC, Zhang SJ, Lanner JT, Wibom R, Katz A, Bruton JD, Westerblad H (2007). Effects of palmitate on Ca(2+) handling in adult control and ob/ob cardiomyocytes: impact of mitochondrial reactive oxygen species. Diabetes.

[CR13] Galloway CA, Yoon Y (2015). Mitochondrial dynamics in diabetic cardiomyopathy. Antioxid Redox Signal.

[CR14] Haag SM, Gulen MF, Reymond L, Gibelin A, Abrami L, Decout A, Heymann M, van der Goot FG, Turcatti G, Behrendt R, Ablasser A (2018). Targeting STING with covalent small-molecule inhibitors. Nature.

[CR15] Isfort M, Stevens SC, Schaffer S, Jong CJ, Wold LE (2014). Metabolic dysfunction in diabetic cardiomyopathy. Heart Fail Rev.

[CR16] Ishikawa H, Ma Z, Barber GN (2009). STING regulates intracellular DNA-mediated, type I interferon-dependent innate immunity. Nature.

[CR17] Jarosz J, Ghosh S, Delbridge LM, Petzer A, Hickey AJ, Crampin EJ, Hanssen E, Rajagopal V (2017). Changes in mitochondrial morphology and organization can enhance energy supply from mitochondrial oxidative phosphorylation in diabetic cardiomyopathy. Am J Physiol Cell Physiol.

[CR18] Konstantinidis K, Kitsis RN (2012). Cardiovascular biology: escaped DNA inflames the heart. Nature.

[CR19] Lam E, Stein S, Falck-Pedersen E (2014). Adenovirus detection by the cGAS/STING/TBK1 DNA sensing cascade. J Virol.

[CR20] Li N, Zhou H, Wu H, Wu Q, Duan M, Deng W, Tang Q (2019). STING-IRF3 contributes to lipopolysaccharide-induced cardiac dysfunction, inflammation, apoptosis and pyroptosis by activating NLRP3. Redox Biol.

[CR21] Li Q, Liu C, Yue R, El-Ashram S, Wang J, He X, Zhao D, Zhou X, Xu L (2019). cGAS/STING/TBK1/IRF3 signaling pathway activates BMDCs maturation following Mycobacterium bovis infection. Int J Mol Sci.

[CR22] Liu S, Feng M, Guan W (2016). Mitochondrial DNA sensing by STING signaling participates in inflammation, cancer and beyond. Int J Cancer.

[CR23] Liu S, Du F, Li X, Wang M, Duan R, Zhang J, Wu Y, Zhang Q (2017). Effects and underlying mechanisms of irisin on the proliferation and apoptosis of pancreatic β cells. PLoS ONE.

[CR24] López-Armada MJ, Riveiro-Naveira RR, Vaamonde-García C, Valcárcel-Ares MN (2013). Mitochondrial dysfunction and the inflammatory response. Mitochondrion.

[CR25] Luo X, Li H, Ma L, Zhou J, Guo X, Woo SL, Pei Y, Knight LR, Deveau M, Chen Y, Qian X, Xiao X, Li Q, Chen X, Huo Y, McDaniel K, Francis H, Glaser S, Meng F, Alpini G, Wu C (2018). Expression of STING is increased in liver tissues from patients with NAFLD and promotes macrophage-mediated hepatic inflammation and fibrosis in mice. Gastroenterology.

[CR26] Ma Z, Damania B (2016). The cGAS-STING defense pathway and its counteraction by viruses. Cell Host Microbe.

[CR27] Maekawa H, Inoue T, Ouchi H, Jao TM, Inoue R, Nishi H, Fujii R, Ishidate F, Tanaka T, Tanaka Y, Hirokawa N, Nangaku M, Inagi R (2019). Mitochondrial damage causes inflammation via cGAS-STING signaling in acute kidney injury. Cell Rep.

[CR28] Marinho FV, Benmerzoug S, Oliveira SC, Ryffel B, Quesniaux VFJ (2017). The emerging roles of STING in bacterial infections. Trends Microbiol.

[CR29] Montaigne D, Marechal X, Coisne A, Debry N, Modine T, Fayad G, Potelle C, El Arid JM, Mouton S, Sebti Y, Duez H, Preau S, Remy-Jouet I, Zerimech F, Koussa M, Richard V, Neviere R, Edme JL, Lefebvre P, Staels B (2014). Myocardial contractile dysfunction is associated with impaired mitochondrial function and dynamics in type 2 diabetic but not in obese patients. Circulation.

[CR30] Morehouse BR, Govande AA, Millman A, Keszei AFA, Lowey B, Ofir G, Shao S, Sorek R, Kranzusch PJ (2020). STING cyclic dinucleotide sensing originated in bacteria. Nature.

[CR31] Mottis A, Herzig S, Auwerx J (2019). Mitocellular communication: shaping health and disease. Science.

[CR32] Ni R, Cao T, Xiong S, Ma J, Fan GC, Lacefield JC, Lu Y, Le Tissier S, Peng T (2016). Therapeutic inhibition of mitochondrial reactive oxygen species with mito-TEMPO reduces diabetic cardiomyopathy. Free Radic Biol Med.

[CR33] Ogurtsova K, Fernandes JD, Huang Y, Linnenkamp U, Guariguata L, Cho NH, Cavan D, Shaw JE, Makaroff LE (2017). IDF Diabetes Atlas: global estimates for the prevalence of diabetes for 2015 and 2040. Diabetes Res Clin Pract.

[CR34] Oka T, Hikoso S, Yamaguchi O, Taneike M, Takeda T, Tamai T, Oyabu J, Murakawa T, Nakayama H, Nishida K, Akira S, Yamamoto A, Komuro I, Otsu K (2012). Mitochondrial DNA that escapes from autophagy causes inflammation and heart failure. Nature.

[CR35] Peterson LR, Gropler RJ (2020). Metabolic and molecular imaging of the diabetic cardiomyopathy. Circ Res.

[CR36] Rijzewijk LJ, van der Meer RW, Smit JW, Diamant M, Bax JJ, Hammer S, Romijn JA, de Roos A, Lamb HJ (2008). Myocardial steatosis is an independent predictor of diastolic dysfunction in type 2 diabetes mellitus. J Am Coll Cardiol.

[CR37] Schilling JD (2015). The mitochondria in diabetic heart failure: from pathogenesis to therapeutic promise. Antioxid Redox Signal.

[CR38] Schulze PC, Drosatos K, Goldberg IJ (2016). Lipid use and misuse by the heart. Circ Res.

[CR39] Seferović PM, Paulus WJ (2015). Clinical diabetic cardiomyopathy: a two-faced disease with restrictive and dilated phenotypes. Eur Heart J.

[CR40] Shimada K, Crother TR, Karlin J, Dagvadorj J, Chiba N, Chen S, Ramanujan VK, Wolf AJ, Vergnes L, Ojcius DM, Rentsendorj A, Vargas M, Guerrero C, Wang Y, Fitzgerald KA, Underhill DM, Town T, Arditi M (2012). Oxidized mitochondrial DNA activates the NLRP3 inflammasome during apoptosis. Immunity.

[CR41] Tanaka Y, Chen ZJ (2012). STING specifies IRF3 phosphorylation by TBK1 in the cytosolic DNA signaling pathway. Sci Signal.

[CR42] Thapa D, Nichols CE, Lewis SE, Shepherd DL, Jagannathan R, Croston TL, Tveter KJ, Holden AA, Baseler WA, Hollander JM (2015). Transgenic overexpression of mitofilin attenuates diabetes mellitus-associated cardiac and mitochondria dysfunction. J Mol Cell Cardiol.

[CR43] Wei WY, Ma ZG, Zhang N, Xu SC, Yuan YP, Zeng XF, Tang QZ (2018). Overexpression of CTRP3 protects against sepsis-induced myocardial dysfunction in mice. Mol Cell Endocrinol.

[CR44] West AP, Shadel GS (2017). Mitochondrial DNA in innate immune responses and inflammatory pathology. Nat Rev Immunol.

[CR45] Westermeier F, Navarro-Marquez M, López-Crisosto C, Bravo-Sagua R, Quiroga C, Bustamante M, Verdejo HE, Zalaquett R, Ibacache M, Parra V, Castro PF, Rothermel BA, Hill JA, Lavandero S (2015). Defective insulin signaling and mitochondrial dynamics in diabetic cardiomyopathy. Biochim Biophys Acta.

[CR46] Xiao Y, Wu QQ, Duan MX, Liu C, Yuan Y, Yang Z, Liao HH, Fan D, Tang QZ (2018). TAX1BP1 overexpression attenuates cardiac dysfunction and remodeling in STZ-induced diabetic cardiomyopathy in mice by regulating autophagy. Biochim Biophys Acta Mol Basis Dis.

[CR47] Zhang C, Shang G, Gui X, Zhang X, Bai XC, Chen ZJ (2019). Structural basis of STING binding with and phosphorylation by TBK1. Nature.

[CR48] Zhao M, Lu L, Lei S, Chai H, Wu S, Tang X, Bao Q, Chen L, Wu W, Liu X (2016). Inhibition of receptor interacting protein kinases attenuates cardiomyocyte hypertrophy induced by palmitic acid. Oxid Med Cell Longev.

[CR49] Zorov DB, Juhaszova M, Sollott SJ (2014). Mitochondrial reactive oxygen species (ROS) and ROS-induced ROS release. Physiol Rev.

