# Supplementary material for: Lipotoxicity-induced mtDNA release promotes diabetic cardiomyopathy by activating the cGAS-STING pathway in obesity-related diabetes
Source: Cell Biol Toxicol. 2022 Mar 2;39(1):277–99. doi: 10.1007/s10565-021-09692-z (PMC10042943; doi:10.1007/s10565-021-09692-z)
Supplement: Supplementary file 1 — Supplementary file1 (DOCX 13 KB) [file 10565_2021_9692_MOESM1_ESM.docx]

| Species | Genes | Forward Primers (5’→3’) | Reverse Primers (5’→3’) |
| --- | --- | --- | --- |
| Mus | cGAS | GAGGCGCGGAAAGTCGTAA | TTGTCCGGTTCCTTCCTGGA |
| Mus | STING | GGTCACCGCTCCAAATATGTAG | CAGTAGTCCAAGTTCGTGCGA |
| Mus | IL-1beta | GTCTTTCCCGTGACCTTC | ATCTCGGAGCCTGTTAGTGC |
| Mus | IL-18 | TCTTGGCCCAGGAACAATGG | ACAGTGAAGTCGGCCAAAGT |
| Mus | mtDNA loop1 | AATCTACCATCCTCCGTGAAACC | TCAGTTTAGCTACCCCCAAGTTTAA |
| Mus | mtDNA loop2 | CCCTTCCCCATTTGGTCT | TGGTTTCACGGAGGATGG |
| Mus | mtDNA loop3 | TCCTCCGTGAAACCAACAA | AGCGAGAAGAGGGGCATT |
| Mus | mtDNA ND4 | AACGGATCCACAGCCGTA | AGTCCTTCGGGCCATGATT |
| Mus | Tert | CTAGCTCATGTGTCAAGACCCTCTT | GCCAGCACGTTTCTCTCGTT |
| Mus | GAPDH | AGGTCGGTGTGAACGGATTTG | TGTAGACCATGTAGTTGAGGTCA |
| Rat | cGAS | CCTCTGCTAGTGTTTTCCGC | GACCGCTGGAAACGAAACTT |
| Rat | STING | GTTACCGCTTCAAACTGGCA | GCACAACTCTTCAGCCAGAC |
| Rat | IL-1beta | GGGATGATGACGACCTGCTAG | CCACTTGTTGGCTTATGTTCTG |
| Rat | IL-18 | CCGCAGTAATACGGAGCA | TGGGATTCGTTGGCTGTT |
| Rat | GAPDH | CCTCAAGATTGTCAGCAAT | CCATCCACAGTCTTCTGAGT |
| Rat | mtDNA loop1 | TGGTTCTTACTTCAGGGCCA | TTTGTGCTGACCTTCATGCC |
| Rat | mtDNA loop2 | GGCATGAAGGTCAGCACAAA | TGGGGTTTGGCATTGAAGTT |
| Rat | mtDNA loop3 | CCTGTCCCCAATTGGTCTCT | AGACCCGTTACCATCGAGAT |
| Rat | mtDNA ND4 | GCCTATCGAGCTTGGTGATA | TATCCTACCTTTGCACGGTC |
| Rat | Tert | CAAGGCCAAGTCCACAAGTC | ACAAAGCGCAGGAAGAAGTG |

**Primers for qPCR**
